# Does usage of monetary incentive impact the involvement in surveys? A systematic review and meta-analysis of 46 randomized controlled trials

**DOI:** 10.1371/journal.pone.0279128

**Published:** 2023-01-17

**Authors:** Basel Abdelazeem, Aboalmagd Hamdallah, Marwa Abdelazim Rizk, Kirellos Said Abbas, Nahla Ahmed El-Shahat, Nouraldeen Manasrah, Mostafa Reda Mostafa, Mostafa Eltobgy

**Affiliations:** 1 McLaren Health Care, Flint, Michigan, United States of America; 2 Michigan State University, East Lansing, Michigan, United States of America; 3 Faculty of Medicine, Al-Azhar University, Damietta, Egypt; 4 Faculty of Medicine, Zagazig University, Ash Sharqia Governorate, Egypt; 5 Faculty of Medicine, Alexandria University, Alexandria, Egypt; 6 Faculty of Medicine for Girls, Al-Azher University, Cairo, Egypt; 7 Detroit Medical Center/Sinai Grace Hospital, Detroit, Michigan; 8 Wayne State University, Detroit, Michigan, United States of America; 9 Rochester Regional/Unity hospital, Rochester, New York, United States of America; 10 The Ohio State University, Columbus, Ohio, United States of America; University of Florida, UNITED STATES

## Abstract

**Background:**

Surveys are an effective method for collecting a large quantity of data. However, incomplete responses to these surveys can affect the validity of the studies and introduce bias. Recent studies have suggested that monetary incentives may increase survey response rates. We intended to perform a systematic review and meta-analysis of randomized controlled trials (RCTs) to evaluate the effect of monetary incentives on survey participation.

**Methods:**

A systematic search of electronic databases was conducted to collect studies assessing the impact of monetary incentives on survey participation. The primary outcome of interest was the response rates to incentives: money, lottery, and voucher. We used the Cochrane Collaboration tool to assess the risk of bias in randomized trials. We calculated the rate ratio (RR) with its 95% confidence interval (95% CI) using Review Manager Software (version 5.3). We used random-effects analysis and considered the data statistically significant with a P-value <0.05.

**Results:**

Forty-six RCTs were included. A total of 109,648 participants from 14 countries were involved. The mean age of participants ranged from 15 to more than 60 years, with 27.5% being males, 16.7% being females, and the other 55.8% not reported. Our analysis showed a significant increase in response rate in the incentive group compared to the control group, irrespective of the incentive methods. Money was the most efficient way to increase the response rate (RR: 1.25; 95% CI: 1.16,1.35; P = < 0.00001) compared to voucher (RR: 1.19; 95% CI: 1.08,1.31; P = < 0.0005) and lottery (RR: 1.12; 95% CI: 1.03,1.22; P = < 0.009).

**Conclusion:**

Monetary incentives encourage the response rate in surveys. Money was more effective than vouchers or lotteries. Therefore, researchers may include money as an incentive to improve the response rate while conducting surveys.

## Introduction

Surveys allow researchers to collect a large quantity of data efficiently. It can be widely applied to collect information like participants’ demographics, knowledge, past behaviors, and opinions. Surveys are a reliable and precise method for data gathering as they provide all the participants with standardized and uniform questions. Surveys can be conducted in different ways, such as written questionnaires, face-to-face or telephone interviews, and online (email or website) surveys.

The generalizability of survey results depends mainly on response rate; refusing to respond to surveys could lead to nonresponse bias which occurs when there is a significant difference between participants who responded to the survey and those who did not. It has a negative effect on the reliability and validity of survey study findings [1]. Achieving adequate response rates is a significant obstacle in survey research. Therefore, efforts are continuously made to improve survey response rates using different methods like incentives. Various incentives have been used to increase response rates, such as candy, lottery, vouchers, and money [2–4].

Non-monetary incentives like shopping vouchers are commonly used to improve survey response, but it has little to no impact on the survey rate. On the other hand, monetary stimulus has successfully increased response rates. For example, one systematic review found the survey response rate doubled upon receiving monetary incentives [5], and another showed that using incentives increased participation in clinical research [6]. We conducted a systematic review and meta-analysis of randomized controlled trials (RCTs) to evaluate the overall effectiveness of incentives like the lottery, vouchers, and money in enhancing response rates to surveys.

## Methods

We followed the Preferred Reporting Items for Systematic Reviews and Meta-Analyses (PRISMA) statement and Cochrane Handbook for Systematic Reviews of Interventions [7, 8] S1 File.

### Data sources and search strategy

We performed a comprehensive and systematic search of the PubMed, Web of Science, Scopus, Embase, and Cochrane library databases from inception to the 23^rd^ of September 2021. A manual updated search was conducted at the end of November 2021. There were no language or publishing date restrictions. A combination of keywords and standardized index terms was used to generate the search strategies. We used keywords like "payments", "incentive", "response", "participation", "enrollment", and "randomized" and their synonyms in our search. The full research strategy and results in different databases are reported in S1 Table.

### Study selection and eligibility criteria

The criteria of study selection we used to select studies in this meta-analysis were as follows: (1) study design: randomized controlled trials;(2) the study investigated the effects of incentive compared to no incentive on participation in surveys;(3) there were no specific criteria to the study participants;(4) the outcome of interest was the response rate. We excluded nonrandomized trials and articles without relevant population, intervention, or outcomes.

Two reviewers (AH and MAR) independently screened and selected studies using the eligibility criteria. Any disagreements were resolved by discussion with third reviewers (KSA and NAE).

### Data extraction

Data were extracted using a formatted excel sheet, including the first author’s last name, year of publication, country, the number of reminders, population criteria, incentive types, sample size, age, and gender. All data were separately extracted by (AH and MAR) and integrated clearly. Disagreements were solved through discussion between reviewers (KSA and NAE).

### Risk of bias assessment

To assess the bias in all included RCTs, two reviewers (AH and MAR) used the Cochrane Collaboration’s tool for assessing the risk of bias in randomized trials [9], which covers biases including selection bias, performance bias, attrition bias, detection bias, reporting bias, and other biases. Each domain’s risk of bias was categorized as high, unclear, or low risk of bias. Any conflicts were resolved by consulting a third reviewer (KSA and BA).

### Outcomes of interest

The primary outcome of interest was the response rates according to the types of incentives: money, lottery, and voucher. Data about response rate was extracted in the format of event/ total for both incentive and control groups.

### Statistical analysis

We used Review Manager Software (version 5.3) to perform the meta-analysis [10]. We calculated the risk ratio (RR) with its 95% confidence interval (95% CI) using event and total numbers. We considered the data statistically significant if the P-value was less than 0.05. We used I^2^ a to evaluate the heterogeneity between the included studies by random-effects analysis. Heterogeneous was considered to be low if I^2^ < 50%. We performed our analysis based on the type of incentive; money, lottery, or voucher. We assessed publication bias throughout the included studies for money used in response rate [11]. A funnel plot was used by plotting the risk ratio (RR) on the x-axis and the log of risk ratio on the y-axis.

## Results

### Study identification and selection

**Fig 1:**
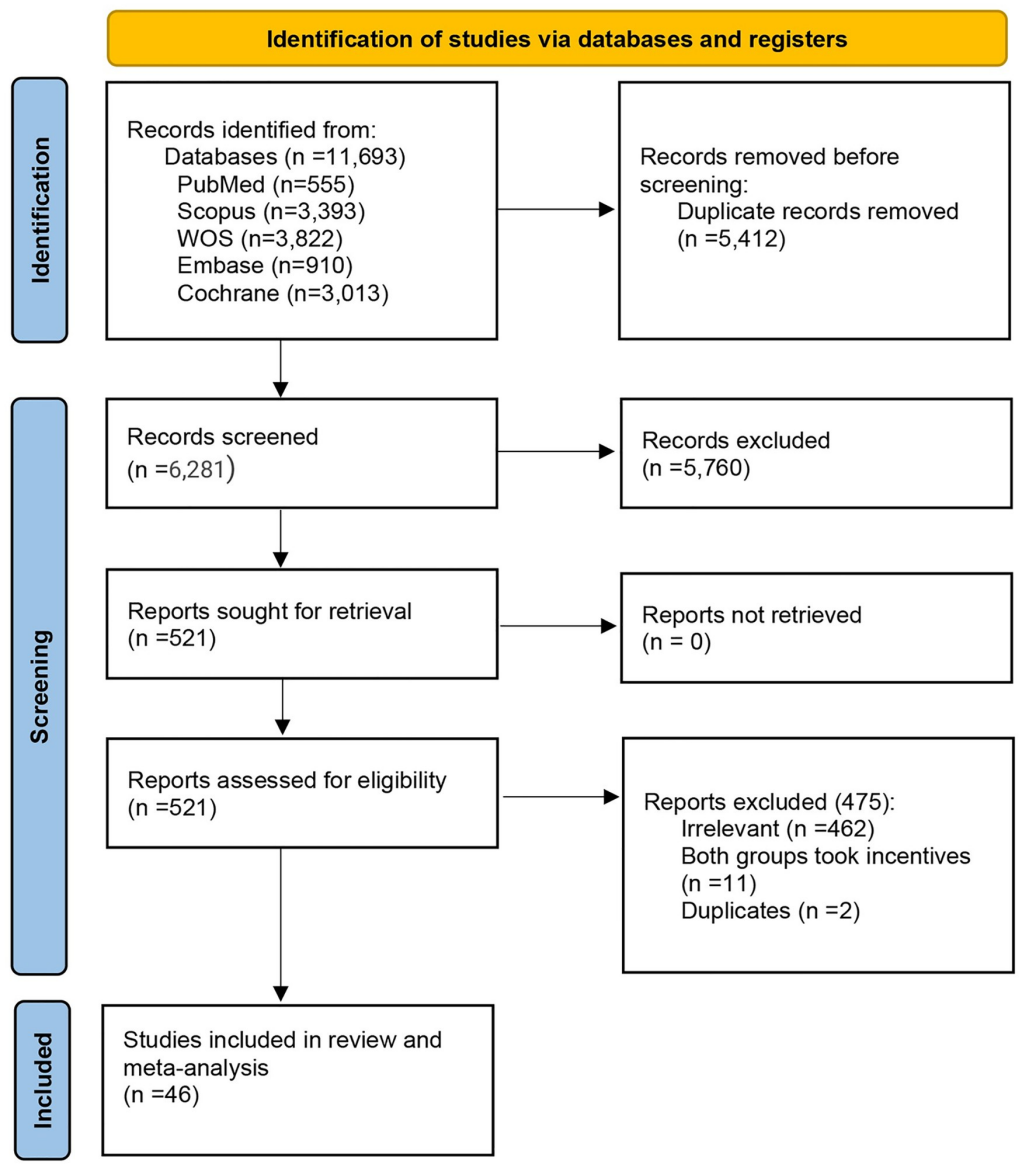
PRISMA 2020 flow diagram for systematic reviews, which included searches of databases, registers, and other sources.

The search retrieved 11,693 articles. After 5,412 duplicates were removed, 6,281 articles were screened. We excluded 5,760 at the title/abstract screening stage as they were not eligible. The remaining 521 articles underwent a full-text evaluation to determine eligibility. Finally, 46 RCTs [3, 4, 12–55] met the criteria for final inclusion in our systematic review and meta-analysis. The process of study selection and the reasons for exclusion are shown in Fig 1.

### Characteristics of included studies

Our search identified 46 studies with 109,648 participants from 14 countries; 27.5% were males, 16.7% were females, and 55.8% were not reported. The average age ranged from 15 to more than 60 years. The duration of the questionnaire ranged from one week to one year. The summary of the included studies and baseline characteristics of participants are shown in Table 1.

**Table 1:** Characteristics of the included studies.

Author, year.	Country	Survey aim and format	Number of reminders	Population criteria	Incentive types	Sample size	Age, year mean (SD) or range (min. to max.)	Gender
								female n (%)
Agarwal et al. 2016 [3]	Canada	Mailed survey assessing how departments measure faculty productivity for the purpose of salary compensation	2	Chairpersons of all academic Departments of Medicine in the United States.	None	76	NR	NR
					$5 Starbucks gift card			
Biner et al. 1988 [12]	India	Mailed survey to assess community needs	NR	The participants were residents of a Midwestern city, selected from the current local telephone directory according to a systematic random-sampling technique.	Non-incentive group	90	NR	NR
					$1 group	88		
Blomberg et al. 1996 [13]	Sweden	Mailed survey to evaluate psychotherapy follow-up	3	The participants were patients who had been in diagnostic interviews at the Stockholm County Council Institute of Psychotherapy three years before the time of the study	Non-incentive group	29	34 (7.5)	85 (100)
					Conditionalincentive group	28		
					Non- Conditionalincentive group	28		
Boucher et al. 2015 [14]	New Zealand	Mail survey about to investigate factors related to the prevention of weight gain	3	Women between the ages of 40–50 at baseline who were able to read and understand English.	Incentive	2,035	40–50	2035 (100)
					Non incentive	289		289 (100)
Chen et al. 2015 [15]	China	Interview based survey about general health	3	Selected Liangping district as the incentive group and Yongchuan district as the control group because the economic development of the two districts is comparable	None	2,799	≥ 15	1399
					Cash incentive	2,662		1327
Cheung et al. 2019 [16]	China.	Interview based survey about smoking	NR	Respondents who self-reported current use of cigarettes were eligible for the RCT.	None	316	≥ 18	51(16.2)
					ConditionalHK$100 incentive	326		49 (15.0)
					ConditionalHK$200 incentive	283		40 (14.1)
					Prepaid HK$100 incentive and ConditionalHK$100 incentive	321		53 (16.5)
Conn et al. 2019 [17]	USA	Web-based survey assessing former applicants to teach for India	NR	College-educated professionals who applied to join teach for India, a not-for-profit service organization in India, between 2009 and 2014.	None	362	NR	NR
					Few large prizes (lottery; two $1,000)	363		
					Many small prizes (lottery; 20 $100)	345		
					Few large and many small prizes (lottery)	345		
					$5 donation to a charity	365		
Coogan et al. 2004 [18]	USA	Telephone interview about colorectal cancer	NR	The participants were Large Bowel Cancer patients.	Incentive group	193	50–70	89 (44.7)
					Non-incentive group	199		87 (45.1)
Coryn et al. 2019 [19]	USA	Web-based survey to investigate whether or not one decides to complete a survey questionnaire	4	AEA members	None	226	NR	557 (61.58)
					Lottery	226		
					Token incentive	226		
					Philanthropic donation	226		
Cottrell et al. 2015 [20]	UK	Mailed survey to to investigate the attitudes, beliefs and reported clinical management of general practitioners regarding exercise for chronic knee pain	2	The participants were general practitioners.	Incentive group	391	NR	NR
					Non-incentive group	390		
Doerfling et al. 2010 [21]	USA	Web-based survey of physical activity and joint health	2	Members of the Canadian Association of Retired Persons	None	575	NR	171 (60.9)
					Entry into a draw	575		
Doody et al. 2003 [22]	USA	Mailed survey to estimate cancer risk among radiologic technologists	NR	Radiologic technologists first certified by the American Registry of Radiologic technologists between 1926 and 1982 non responders to the initial survey	None (referent)	595	NR	407(68.4)
					1$ bill	590		392(66.4)
					2$ bill	589		398(67.6)
					2$ check	592		430(72.65)
					5$ check	298		199(66.8)
Drummond et al. 2014 [23]	Ireland	Mailed survey to seek information on PCP demographics, training, practice size and mix	2	The participants selected through a postal questionnaire of PCPs’ practice and costs in relation to prostate-specific antigen testing in Ireland.	Non-incentive group	459	< 40	0
					Prize group	484		
					Cash group	478		
Dykema et al. 2011 [24]	USA	Web based survey to assess physicians’ knowledge of human genetic variation and their use of patient’s characteristics	4	The physician currently practicing, board-certified with internal medicine as their primary specialty, and reside in the United States. Physicians were eligible if they had both an email and postal address on file	None	1,500	NR	1,140 (32.1)
					Conditional$200 lottery	1,000		
					Conditional$50 check	700		
					Conditional$100 check	350		
Everett et al. 1997 [25]	USA	Mailed survey to assess the family physicians’ firearm-safety counseling beliefs and pratices	2	Primary care physicians that lived in USA at the time of mailing	None	300	NR	NR
					1$ bill	300		
Friedman et al. 1979 [26]	USA	Mailed survey about public’s attitudes towards various etchnic groups	NR	The participants were selected from the new telephone directories of two suburban areas in the greater New York Metropolitan area.	Incentive group	150	NR	NR
					Non-incentive group	150		
Gajic et al. 2012 [27]	Canada	Web-based survey of a general community population	1	Community members	None	705	58.4 (12.6)	13 (33%)
					Prepaid incentive $2	705	48.7 (14.3)	55 (45%)
					Low lottery (10 x $25)	694	52.5 (13.6)	13 (33%)
					High lottery (2 x $250)	709	50.6 (13.4)	31 (41%)
Gates et al. 2009 [28]	UK	Mailed survey about managing injuries of the neck	NR	The participants were from Managing Injuries of the Neck Trial, they are included 4–8 months after their whiplash injury.	£5 gift voucher	1,070	37 (13.4)	613 (57.3)
					No gift voucher	1,074	36.8 (13.2)	606 (56.4)
Gjøstein et al. 2016 [29]	Norway	Mailed survey on risk factors for colorectal cancer	1	Women and men aged 55–64 years and resident in Aust-Agder or Vest-Agder counties	None	2,264	55–64	1 963 (53.0)
					Unconditional scratch card	2,267		
					Conditional prize draw for ipad	2,264		
Glenny et al. 2013 [30]	UK.	Mailed survey about the dental health of young children	NR	Any school, with 20 or more 5- year-old children participating in the Dental Health Survey within these PCTs.	None	66	NR	NR
					Financial incentive	127		
Glidewell et al. 2012 (RCT A) [31]	UK	Mailed survey of health professional	2	The participants were dentists in Scotland, who had not been invited to participate in the first PRIME study.	Incentive group	102	NR	NR
					Non-incentive group	102		
					Burden group	102		
Guo et al. 2016 [32]	Canada	Various survey types that asked about general health, quality of life, and use of health services, with an emphasis on osteoarthritis.	NR	Households in British Columbia	None	1,000	53.4 (16.0)	NR
					Instant lottery	1,000	51.0 (16.5)	
					$2 prepaid coin incentive	1,000	50.4 (16.4)	
					Prepaid Coin incentive + instant lottery	1,000	51.9 (15.4)	
Hall et al. 2019 [33]	USA	Online survey about HIV prevention and risk behaviors.	NR	Eligible men lived in the United States, reported being male at birth, reported being between 18–34 years old and reported having anal or oral sex with one or more men in the past year.	None	285	18–34	0% female
					Monetary incentive ($20 gift card)	355		
Hawley et al. 2010 [34]	USA	Mailed survey of mental health assessment and treatment practices.	Up to three mailings (initial survey with incentive, postcard, follow-up survey)	500 mental health providers randomly selected from guild membership lists, 100 from each of five professional organizations (ACA; AAMFT;AACAP; APA; NASW).	None	98	NR	NR
					1$ prepaid	100		
					2$ prepaid	99		
					5$ prepaid	99		
Jacob et al. 2012 [35]	USA	Varied modes of survey delivery to assess school principals’ participation in educational research	2	The participants were school principals using online learning in Michigan high schools.	$10 Incentive group	602	NR	185 (30.7)
					Non-incentive group	575		205 (35.7)
John et al. 1994 [36]	USA	Mailed survey to identify women with a recent pregnancy	3	The participants were females’ cosmetologists between 22 and 36 years old who were licensed in North Carolina in April 1988.	$1 enclosed with first mailing	2,791	24–36	2791 (100)
					$1 enclosed with second mailing	4,315		4315 (100)
					No monetary incentive enclosed	443		443 (100)
Kenyon et al. 2005 [37]	UK	Postal survey to assess the health and development of children	1	The participants were parents of children enrolled in The MRC ORACLE Children Study.	Voucher	369	NR	NR
					No voucher	353		
Knoll et al. 2012 [38]	Canada	Telephone survey to estimate the prevalence of food allergy	NR	The participants were low socioeconomic status, low education or new Canadians.	Incentive group	364	18 years or older	NR
					Non-incentive group	364		
Kypri et al. 2016 [39]	Australia	Survey pack about alcohol	NR	Residents on the New Zealand General and Maori electoral rolls	None	1,600	NR	NR
					Entry into a $500 supermarket voucher prize	2,400		
Moses et al. 2004 [40]	UK	Mailed questionnaires to assess the national consultant obstetricians and gynaecologists	1	All current consultants identified from the RCOG database	None	694	NR	NR
					Prize draw incentive	716		
O’Conner et al. 2011 [41]	Denmark	Mailed questionnaire measuring depression, social support, coping style, adult attachment, life satisfaction, and personality factors	2–3	The participants were elderly married people selected through the Danish Central Person Register.	Full questionnaire with the general initiatives.	300	65–80	NR
					Short questionnaire of a non-sensitive nature and with the general initiatives.	300		
					Full questionnaire sent with recorded response and with the general initiatives.	300		
					Full questionnaire enclosed a gift voucher of 50 dkr./ $10 as a token of appreciation not conditional of response, and with the general initiatives.	300		
Oden et al.1999 [42]	USA	Mailed survey to assess nurse practitioners’ perceived self-efficacy regarding their involvement in public policy	1	The participants were certified nurses from 48 states.	Incentive group	300	43.8 (8.3)	558 (93)
					Non-incentive group	300		
Olsen et al. 2012 [43]	Norway	Mailed questionnaires about oral health	1	The questionnaire was mailed to random population samples in Norway.	Incentive group	1,200	21–60	NR
					Non-incentive group	1,200		
Paolillo et al. 1984 [44]	USA	Mailed survey to assess response rate	NR	The participants were expert selected from employees, executives of financial institutions, state representatives and senators, and executive directors of chambers of commerce.	Non-incentive group	100	NR	NR
					$1.00 enclosed incentive	100		
					$2.00 promised incentive	100		
					Lottery promised	100		
Parsons et al. 2013 [45]	USA	Web—based survey to investigate the influence of various campus housing amenities and student characteristics on academic outcomes	2	The participants were first-year students who lived in on-campus housing.	Non-incentive group	298	18–19	162 (36)
					$2 group	156		
Perneger et al. 1993 [46]	Switzerland	Mailed survey about epidemiologic health	1	The participants who transformed from the University of Geneva group health insurance into a managed health care plan.	No incentive	311	25–36	147 (47)
					Reminder card	309		144(47)
					Money offer	310		150(48)
					Money offer and reminder card	305		158(52)
Robb et al. 2017 [47]	UK	Mailed survey about health psychology	NR	General Practices in South-East England aged 45–59	None	1,033	45–59	1996(47)
					£2.50	1,031		
					£5.00	1,007		
					£250 prize draw	986		
Roberts et al. 2000 [48]	England	Mailed survey about menopause services	2	Women aged 40 to 65 during September to November 1997	None	374	40–65	374 (100)
					Payment	125		125 (100)
					Lottery	374		374 (100)
					Payment + lottery	125		125 (100)
Spry et al. 1989 [49]	USA	Survey contained eight pages of questions on health and physical activity habits, along with some social and developmental variables	NR	The participants selected from telephone directory.	Lottery	200	43–55	142 (35.6)
					No lottery	200		
Takao et al. 2021 [4]	Japan	Online survey to assess the participation in dementia prevention activities and to provide support to people with dementia	NR	The participants were Japanese aged ≥60 years who were recruited by being a registered member of Cross Marketing.	No rewards	750	60–69	375
					Cash rewards			
					Time credits			
Trussell et al. 2004 [50]	USA	Mailed survey to assess the cooperation rates of sending television viewing diaries and material.	NR	Randomly assigned households	None	1,930	NR	NR
					1$	896		
					2$	1,634		
					3$	2,132		
					4$	6,759		
					5$	7,085		
					6$	6,997		
					7$	2,408		
					8$	1,096		
					10$	692		
Turnbull et al. 2015 [51]	USA	Web-based survey to the intensive care physicians to choose the value of compensation for participation	1	The participants were physicians from US hospitals with training programs accredited by the Accreditation Council for Graduate Medical Education in Internal Medicine-Critical Care Medicine, Anesthesiology-Critical Care Medicine, and Surgical Critical Care.	Incentive group	859	NR	156 (18.2)
					Non-incentive group	991	NR	205 (20.7)
Whiteman et al. 2003 [52]	USA	Mailed questionnaires to collect data on women’s health	1	The participants were midlife women in the Baltimore metropolitan region who reported their history of hot flashes and other information through a mailed questionnaire.	Lottery/postcard	298	40–60	298 (100)
					Money/postcard	299		299 (100)
					Postcard only	150		150 (100)
					Lottery only	902		902 (100)
					Money only	900		900 (100)
					No incentive/no postcard	450		450 (100)
Wilson et al. 2010 [53]	UK	Web-based survey via an embedded URL about public health and health services and how	5	The participants were university based and easily use with Internet and emails.	Pre-knowledge about incentive	244	NR	NR
					No knowledge group	241		
Young et al. 2015 [54]	Australia	Web-based survey about cancer care	3	GPs in NSW and Victoria	None	1,122	NR	441(39)
					Unconditional incentive	1,101		401(36)
					Conditional	1,111		430(39)
Zagorsky et al. 2008 [55]	USA	National Longitudinal survey via interview to track women socioeconomic development	NR	The participants were mature and Young Women in the late 1960s	Control group	357	30–44	357 (100)
					$20 group	354		354 (100)
					$40 group	361		361 (100)

NR: not reported, USA: United States of America, UK: United Kingdom, RCT: Randomized controlled trials; AEA: American Evaluation Association; PCP: primary care physician; PCTs: Primary Care Trusts; ACA: American Counseling Association; AAMFT: American Association for Marriage and Family Therapy; AACAP: American Academy of Child and Adolescent Psychiatrists; APA: American Psychological Association; NASW: National Association of Social Workers; MRC: Medical Research Council; RCOG: Royal College of Obstetricians and Gynecologist; GPs: general practitioners: NSW: New South Wales.

### Risk of bias of the included studies

**Fig 2:**
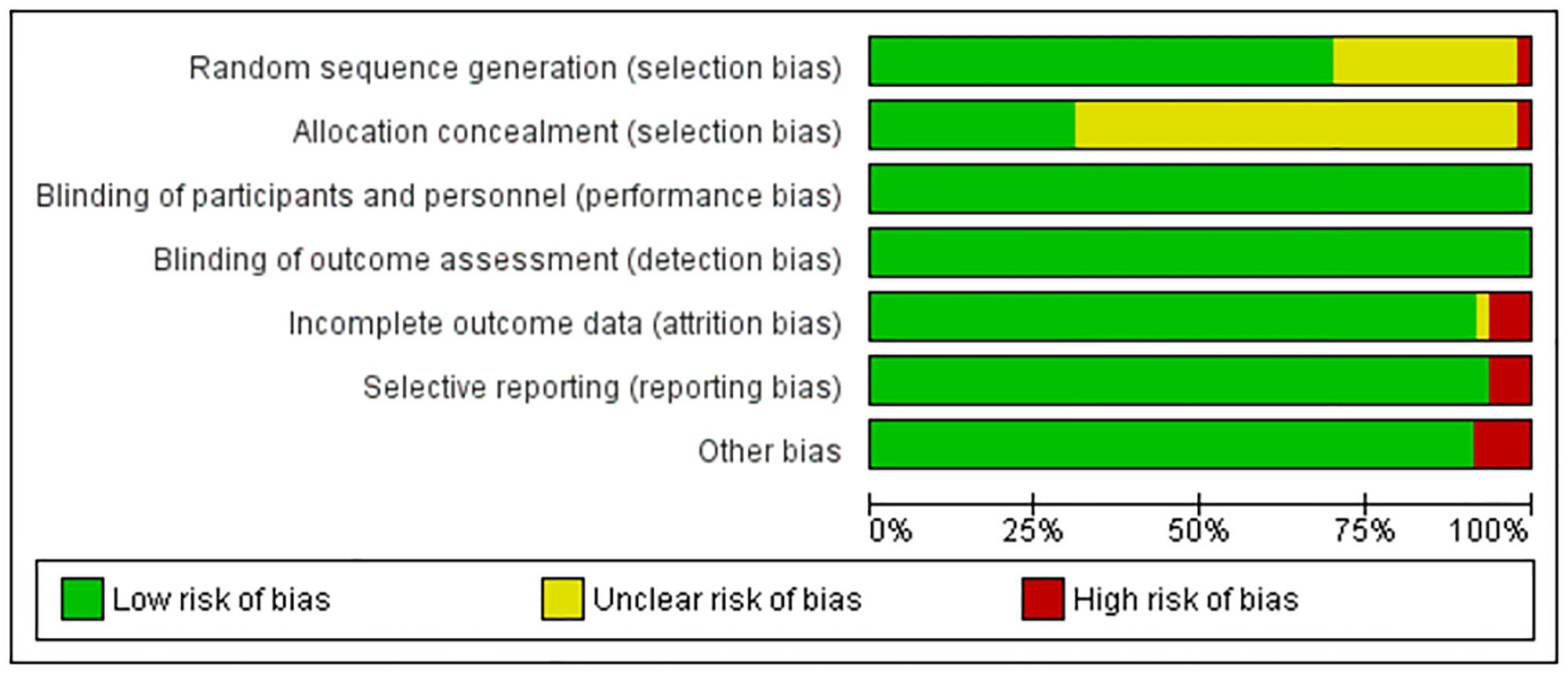
Risk of bias graph: Review authors’ judgments about each risk of bias item presented as percentages across all included studies.

All of the included RCTs demonstrated a low risk of bias in performance and detection biases. Attrition bias was a high risk of bias in three RCTs [33, 35, 41], unclear risk of bias in Boucher et al. [14], and low risk of bias in the rest of the included RCTs. Selective reporting was low risk in all included RCTs, except three RCTs were at high risk of bias [4, 28, 35]. The risk of bias summary for each study and the risk of bias graph for bias domains is shown in Fig 2 and S1 Fig.

### Outcome of interest

**Fig 3:**
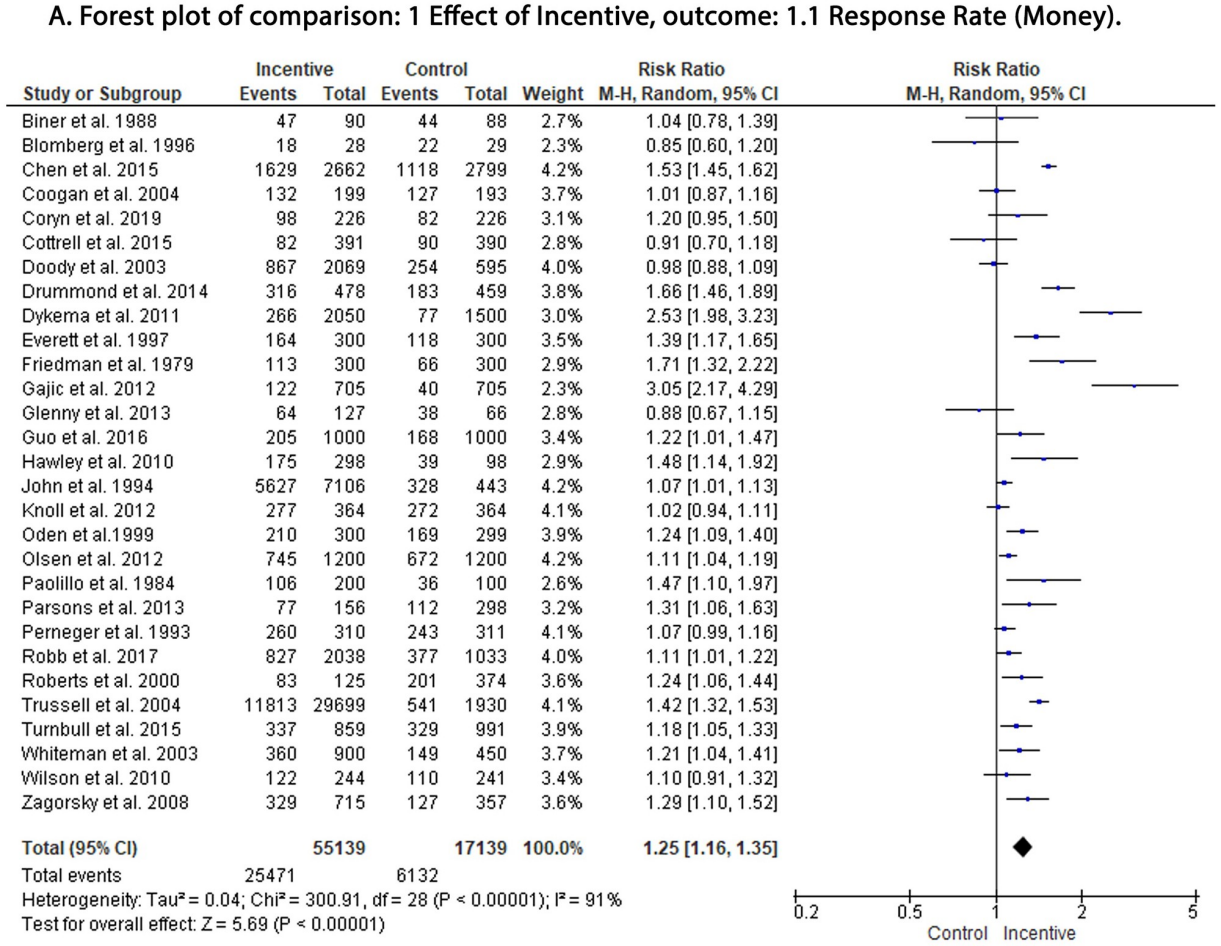
Forest plot of the effect of money in response rate. df: degrees of freedom; I^2^: I-squared; M-H: Mantel-Haenszel variance; CI: confidence interval.

**Fig 4:**
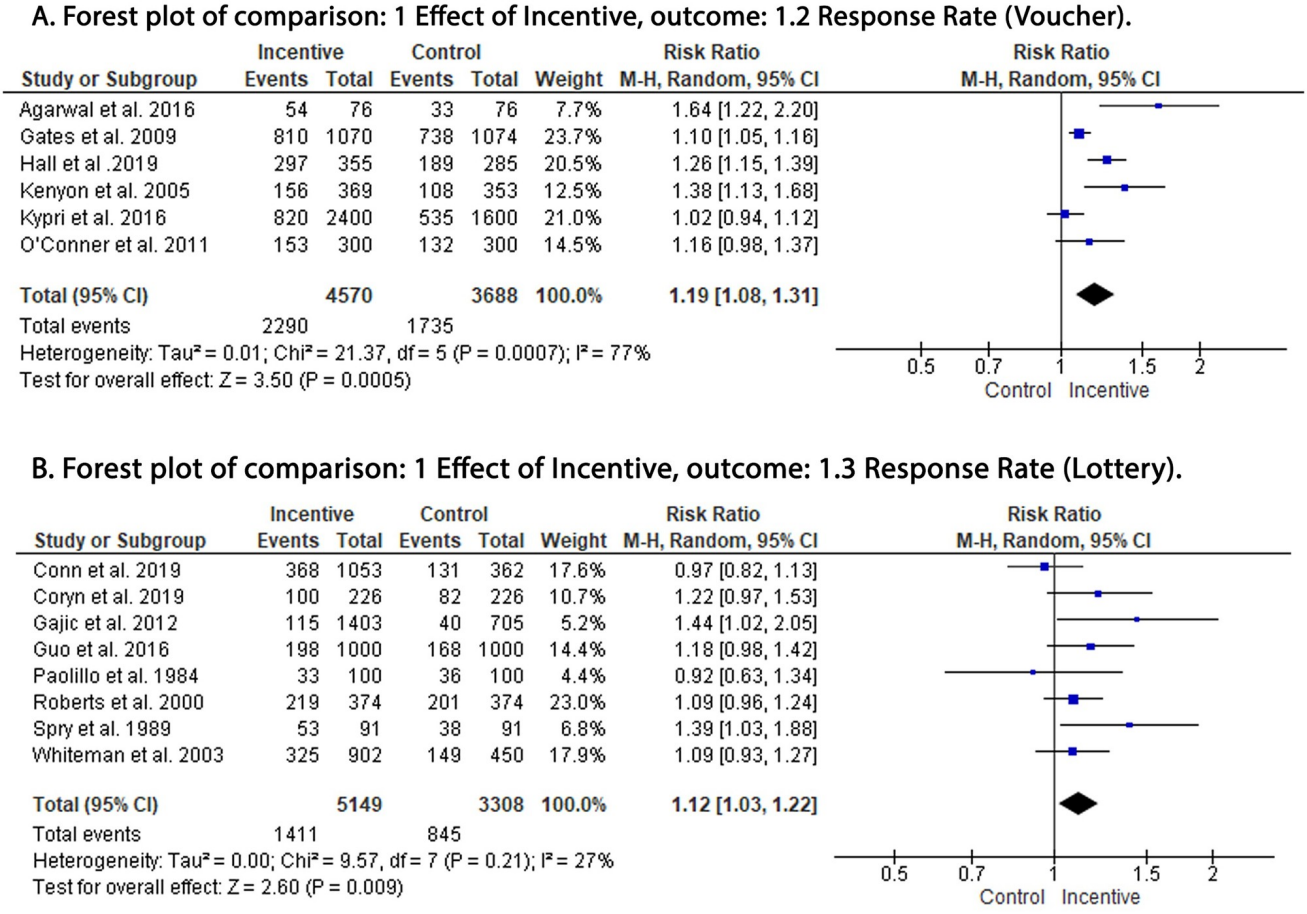
Forest plot of the effect of voucher and lottery in response rate. A: voucher; B: lottery. df: degrees of freedom; I^2^: I-squared; M-H: Mantel-Haenszel variance; CI: confidence interval.

**Fig 5:**
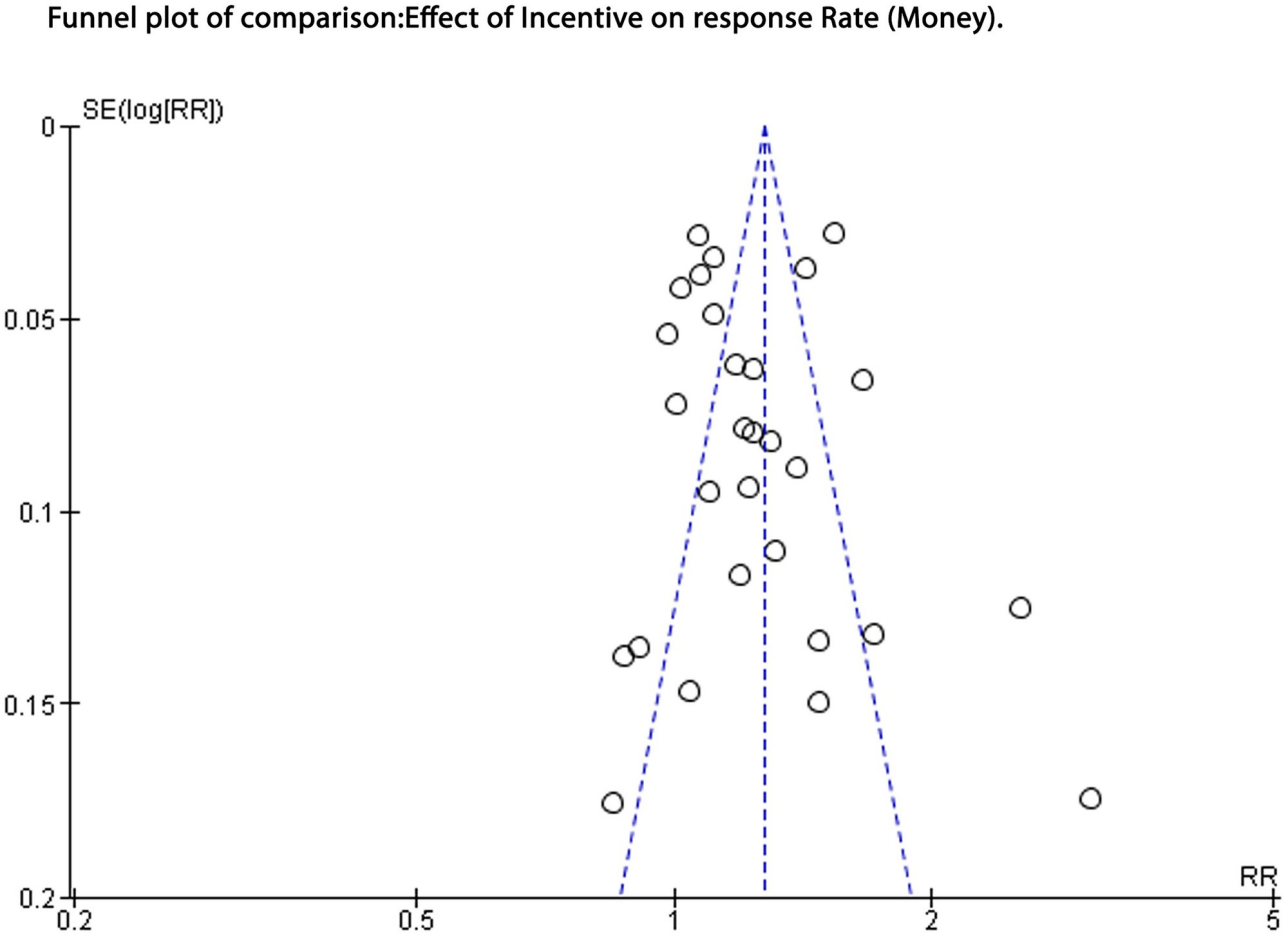
Funnel plot of the publication bias for the effect of money on the response rate. RR: risk ratio.

Our analysis showed a significant increase in response rate in the incentive group compared to the control group, irrespective of the incentive methods. Money was associated with the highest increase in response rate (RR: 1.25; 95% CI: 1.16,1.35; P = < 0.00001) (Fig 3) compared to voucher (RR: 1.19; 95% CI: 1.08,1.31; P = < 0.0005) (Fig 4A) and lottery (RR: 1.12; 95% CI: 1.03,1.22; P = < 0.009) (Fig 4B). The funnel plot for the publication bias had the traditional conal appearance with good bilateral symmetry. Outliers were removed from the analysis and did not affect the pooled RR, suggesting minimal publication bias (Fig 5).

## Discussion

We included 46 RCTs to evaluate the effect of using monetary incentives to increase the participants’ response rate to the survey. Our results showed that using the incentive increased the response rate to surveys. Money was the most effective way, followed by vouchers and lottery.

Monetary incentives have been widely used to enhance response rates to questionnaires, mainly postal surveys. A large systematic review using mailed questionnaires reported that the odds of response to the questionnaire were doubled by using a monetary incentive [56]. Other factors that improve post-mail surveys response include short questionnaires, colored ink, personalized letters, certified mail with a return receipt, and follow-up contact [56]. However, the available evidence on using monetary incentives in electronic/online surveys is limited [57]. Few RCT studies reported that monetary incentives boost the response rate in online surveys compared to no incentives [21, 45, 54]. Surprisingly, a study found that other factors could be as effective as a monetary incentive in increasing the response rate to online surveys, such as well-written short questions, easy accessibility, and engaging topics [19].

Online surveys have a lower response rate compared to postal surveys [58, 59] for several reasons; enrollment of the study sample is challenging compared to postal surveys due to the unavailability of email addresses for all potential invitees and difficulty reaching certain types of participants such as those who do not have internet access [60] also trust is a large obstacle for internet survey participation as invitees may be hesitant to respond because of fears of potential scams, or malicious links infected with computer viruses [61]. However, online surveys have potential advantages by reducing printing and postage costs, branching questions, and adhering to a particular question format (e.g., selecting only one) [62]. Although internet surveys are less effective than other survey methods, combining multiple-mode surveys (e.g., Internet and mail) may improve overall survey response rates [63].

Prepaid incentives (unconditional incentives) are more effective in increasing response rates in comparison to payment after survey completion (conditional incentives) [64, 65]. This could be explained by social exchange theory as providing participants monetary incentives in advance encourages them because they feel they should reciprocate for the reward they receive by completing the survey. On the other hand, promising a gift or money to invitees only after they answer survey questions does not give them the urge to accept the survey invitation [62]. A meta-analysis of 238 experiments concluded that prepaid cash rewards significantly increased the survey response and completion rate compared to contingent monetary incentives upon completion or return of the survey [66].

The prepaid incentive might be challenging to achieve in a web survey, as it is difficult to couple the incentive with the survey request, as reported by [67]. Young et al. confirmed that unconditional incentives had the most significant effect, but they reported that the conditional approach was more cost-effective [54]. A more recent RCT by Cheung et al. found that combining prepaid and promised incentives (mixed incentives) was superior to the promised incentive by increasing the retention rate by 48% [16].

The correlation between incentive value and response rate remains unclear. A systematic review of trials suggests a non-linear relationship between the size of the incentive and the improvement in response [68]. All incentives resulted in a higher response rate than no incentive; the US$2 amount was the most cost-effective. However, the US$10 incentive achieved the highest response rate. Also, cash was more effective than other incentives like prize draws or lotteries [16].

Incentives may enhance response rates and reduce the likelihood of nonresponse error [23]; nevertheless, few studies claim that higher response rates do not necessarily indicate a reduction in nonresponse bias [62, 69]. In addition to that, concerns that incentives may actually introduce response bias by being more appealing to those with lower socioeconomic status [38, 45]. Also, previous research found that women are more likely than men to participate in surveys, so using monetary incentives may exacerbate the overrepresentation of women, raising the risk of response bias. Further studies are required to test the effect of incentives on response and nonresponse bias, plus analyze the impact of different reward sizes on response rates.

Incentives may affect the survey response quality. For example, motivating members to respond who otherwise would have refused will decline response quality and jeopardize the survey outcomes. On the other hand, offering incentives will lead to better quality answers and hence improve survey outcomes. Based on the currently available studies, offering incentives are unlikely to change or alter the quality of the survey [70].

There are a few limitations to our study. Most of the included studies were performed in developed countries like the United States, the United Kingdom, and European countries. The applicability of the results in developing countries needs to be confirmed by enrolling more studies. Allocation concealment bias was unclear in most of the included RCTs, which can introduce bias into our results. We only investigated the effect of incentives on the response rate, but we did not examine the accuracy and reliability of the data collected. More studies are needed to evaluate the effect of incentives on the quality of response. We did not evaluate the relationship between the amount of the incentive offered and the response rate or if there is any minimum amount of monetary incentive that should be other to the participant.

## Conclusions

In conclusion, using monetary incentives is associated with increasing the response rate while conducting a survey. Using money was associated with a higher response rate than vouchers or lottery. Therefore, researchers should include money as an incentive to increase the response rate to the survey.

## Supporting information

S1 File PRISMA 2020 checklist. Preferred Reporting Items for Systematic Review and Meta-Analysis.
(DOCX)

S1 Table Search terms and results in different databases. (DOCX)

S1 Fig Risk of bias summary. Review authors’ judgments about each risk of bias item for each included study. The items are scored (+) low risk; (−) high risk; (?) unclear risk of bias.
(TIF)
