# Supplementary material for: Does usage of monetary incentive impact the involvement in surveys? A systematic review and meta-analysis of 46 randomized controlled trials
Source: PLoS One. 2023 Jan 17;18(1):e0279128. doi: 10.1371/journal.pone.0279128 (PMC9844858; doi:10.1371/journal.pone.0279128)
Supplement: S1 Table — (DOCX) [file pone.0279128.s002.docx]

| Database | Search term | Results |
| --- | --- | --- |
| PubMed | (randomized[Title/Abstract] OR randomised[Title/Abstract] OR random[Title/Abstract] OR randomly[Title/Abstract] OR randomization[Title/Abstract] OR randomisation[Title/Abstract] OR RCT[Title/Abstract] OR RCTs[Title/Abstract]) AND (Payments[Title/Abstract] OR incentive[Title/Abstract]) AND (response[Title/Abstract] OR particip*[Title/Abstract] OR enroll*[Title/Abstract]) | 555 |
| Web of science | (Payments OR incentive) AND (response OR particip* OR enroll*) AND (randomized OR randomised OR random OR randomly OR randomization OR randomisation OR RCT OR RCTs) | 3,822 |
| Scopus | TITLE-ABS ( payments OR incentive ) AND TITLE-ABS ( response OR particip* OR enroll* ) AND TITLE-ABS ( randomized OR randomised OR random OR randomly OR randomization OR randomisation OR rct OR rcts ) | 3,393 |
| Embase | #4: #1 AND #2 AND #3, Results: 910 #1: payments OR incentive, Results: 33,759  #2: response OR participation OR enrollment, Results: 4,211,117  #3: randomized OR randomised OR random OR randomly OR randomization OR randomisation OR rct OR rcts, Results: 1,963,802 | 910 |
| Cochrane | randomized OR randomised OR random OR randomly OR randomization OR randomisation OR rct OR rcts in All Text AND response OR participation OR enrollment in All Text AND payments OR incentive in All Text | 3,013 |

Table S1. Search terms and results in different databases
