# Supplementary figures and images for: Does usage of monetary incentive impact the involvement in surveys? A systematic review and meta-analysis of 46 randomized controlled trials
Source: PLoS One. 2023 Jan 17;18(1):e0279128. doi: 10.1371/journal.pone.0279128 (PMC9844858; doi:10.1371/journal.pone.0279128)

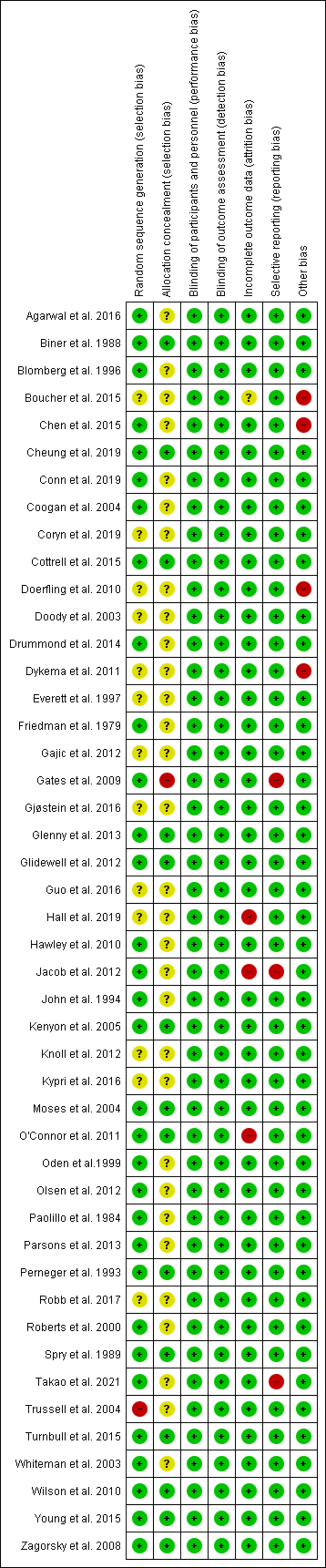

Supplement: S1 Fig — Review authors’ judgments about each risk of bias item for each included study. The items are scored (+) low risk; (−) high risk; (?) unclear risk of bias. (TIF) [file pone.0279128.s003.tif]
